# Effects of hydrogen-rich saline in neuroinflammation and mitochondrial dysfunction in rat model of sepsis-associated encephalopathy

**DOI:** 10.1186/s12967-022-03746-4

**Published:** 2022-11-26

**Authors:** John Sieh Dumbuya, Siqi Li, Lili Liang, Yanchen Chen, Jiang Du, Qiyi Zeng

**Affiliations:** Department of Paediatrics, Zhujiang Hospital of Southern Medical University, Guangzhou, 510282 People’s Republic of China

**Keywords:** Sepsis-associated encephalopathy, Paediatrics, Neuroinflammation, Mitochondrial dysfunction, Apoptosis, Neuronal injury, Astrocyte, Microglia

## Abstract

**Background:**

Sepsis-associated encephalopathy (SAE) is one of the most common types of sepsis-related organ dysfunction without overt central nervous system (CNS) infection. It is associated with higher mortality, low quality of life, and long-term neurological sequelae in suspected patients. At present there is no specific treatment for SAE rather than supportive therapy and judicious use of antibiotics, which are sometimes associated with adverse effects. Molecular hydrogen (H2) has been reported to play crucial role in regulating inflammatory responses, neuronal injury, apoptosis and mitochondrial dysfunction in adult models of SAE. Here we report the protective effect of hydrogen-rich saline in juvenile SAE rat model and its possible underling mechanism(s).

**Materials and methods:**

Rats were challenged with lipopolysaccharide (LPS) at a dose of 8 mg/kg injected intraperitoneally to induce sepsis and hydrogen-rich saline (HRS) administered 1 h following LPS induction at a dose of 5 ml/kg. Rats were divided into: sham, sham + HRS, LPS and LPS + HRS. At 48 h, rats were sacrificed and Nissl staining for neuronal injury, TUNEL assay for apoptotic cells detection, immunohistochemistry, and ELISA protocol for inflammatory cytokines determination, mitochondrial dysfunction parameters, electron microscopy and western blot analysis were studied to examine the effect of HRS in LPS-induced septic rats.

**Results:**

Rats treated with HRS improved neuronal injury, improvement in rats’ survival rate. ELISA analysis showed decreased TNF-α and IL-1β and increased IL-10 expression levels in the HRS-treated group. Apoptotic cells were decreased after HRS administration in septic rats. The numbers of GFAP and IBA-1positive cells were attenuated in the HRS-treated group when compared to the LPS group. Subsequently, GFAP and IBA-1 immunoreactivity were decreased after HRS treatment. Mitochondrial membrane potential detected by JC-1 dye and ATP content were decreased in septic rats, which were improved after HRS treatment, while release of ROS was increased in the LPS group reverted by HRS treatment, ameliorating mitochondrial dysfunction. Further analysis by transmission electron microscopy showed decreased number of mitochondria and synapses, and disrupted mitochondrial membrane ultrastructure in the LPS group, while HRS administration increased mitochondria and synapses number.

**Conclusion:**

These data demonstrated that HRS can improve survival rate, attenuate neuroinflammation, astrocyte and microglial activation, neuronal injury and mitochondrial dysfunction in juvenile SAE rat model, making it a potential therapeutic candidate in treating paediatric SAE.

## Introduction

Sepsis is a life-threatening organ dysfunction caused by a dysregulated host response to infection [1, 2]. Severe infection in children leads to cardiovascular and/or noncardiovascular organ dysfunction referred to as “sepsis-associated organ dysfunction” [3]. Sepsis-induced inflammatory response results in disseminated cerebral dysfunction termed “sepsis-associated encephalopathy, (SAE)” [4], which is characterized by various clinical or laboratory abnormalities in the absence of central nervous system infection, such as abnormal cerebral anatomical structures, haemorrhage or embolism. The incidence of SAE in paediatrics has not been demonstrated [5]. SAE has a long-term impairment, such as behavioral changes, memory and cognitive dysfunction, with socioeconomic burdens on patients/parents and society [6]. In children, the short-term mortality reported are cognitive impairment and poor academic performance [7]; the long-term include delayed neurodevelopment, low verbal IQ, a decline in school performance and low intelligence at short-term follow-up [8].

Manifestations of SAE include seizures, delirium, focal cognitive deficits, hallucinations, abnormal sleep rhythms, personality changes, lack of concentration, and depressive symptoms [9–11]. At present, there are no diagnostic criteria and risk factor stratification for suspected patients with SAE. In other words, direct CNS infection or other types of encephalopathy have to be excluded from diagnosing SAE [12, 13], which hinders its early detection and appropriate implementation of management protocols, thus associated with high mortality rate. This scenario becomes more evident in paediatric patients with few cases being reported in the literature. Clinical assessment, electrophysiological, neurological imaging and biomarkers are implemented to aid diagnosis and direct therapeutic strategies; though most of these diagnostic tools are potentially hampered by sedation, and mechanical ventilation [14–16].

SAE treatment is mainly focused on managing the underlying conditions, as there is no specific treatment protocol [11]. Antibiotics and supportive therapy are the mainstays of treatment, while the sedative medication is used to treat those showing agitation features [10]. Dexmedetomidine [9, 13], therapeutic plasma exchange (TPE) [12, 17], activated protein C [18], etc. have been used to treat patients with suspected SAE. Despite these signs of progress being made, effective treatments are lacking in suspected SAE individuals, especially in children. Thus, other agents are being explored both in vivo and ex vivo which are showing positive and promising results.

The hydrogen molecule is a small (molecular weight 2 Da), electrically neutral, and nonpolar, which is colorless, odorless, diatomic gas produced by intestinal bacteria in mammals. Molecular hydrogen (H2) can easily penetrate several barriers, such as the blood–brain barrier, the placental barrier, and the testis barrier [19–21]. H2 is permeable to biomembranes and can easily target subcellular organelles, especially the mitochondria that constitute the biggest source of cellular ROS [22]; it is non-toxic, balances the pH of body fluids and does not affect the normal metabolic redox reaction due to its small molecular weight and antioxidative activity which selectively affects only the strongest oxidant [23]. It is neither inflammable nor explosive at low concentrations (< 4.6% in air and 4.1% in pure oxygen) [24]. Molecular hydrogen promotes cell detoxification, increases cell hydration, and strengthens the host immune system [25]. H2 consumption can improve biomarkers of inflammation and redox homeostasis [19]. H2 also attenuates injury and dysfunction of important organs (heart, liver, lung, kidneys, and brain) and physiological barriers (epithelial cell barrier, vascular endothelial cell barrier) by suppressing oxidative stress and inflammation as well as reducing apoptosis and regulating sepsis-induced autophagy [25].

Extensive studies both in vivo and ex vivo have shown that H2 play an effective therapeutic role in many disease entities such as sepsis, ischaemia–reperfusion injury, hypoxic-ischemic brain damage (HIBD), organ transplantation, stroke, MODS, type 2 diabetes, atherosclerosis, bronchopulmonary dysplasia (BPD), neurodegenerative diseases, skin diseases, cancers, ionising radiation and oxygen toxicity [20, 25–27]. From the aforementioned, it is perceivable that H2 has multiple roles not only in the CNS but also in other systems by regulating a myriad of mediators and factors activated following brain injuries, such as attenuating neuroinflammation [22, 28, 29], decreasing oxidative stress parameters [30, 31], ameliorating inflammatory response and neuronal apoptosis [32, 33], regulation of signaling pathways [34, 35], improving mitochondrial dysfunction [36, 37], regulating astrocyte and microglial activation [38]. However, there is limited experimental evidence of hydrogen-rich saline (HRS) regulating inflammatory cytokines, neuronal injury, apoptotic cascades and mitochondrial dysfunction in paediatric patients with SAE.

In this study, we investigate the effect of HRS in regulating neuronal apoptosis, mitochondrial dysfunction and neuroinflammation in a juvenile SAE rat model, and the possible underlying mechanism(s). We hypothesized that the administration of HRS following LPS-induced sepsis may attenuate neuroinflammation, astrocyte and microglia activation, mitochondrial dysfunction and neuronal apoptosis, potentially improving overall neurological outcomes.

## Materials and methods

### Cell culture

SH-SY5Y cell lines were grown in Dulbecco’s Modified Eagle Medium (DMEM) supplemented with 5% fetal bovine serum (Gibco), 10 U/ml penicillin, 10 U/ml streptomycin and 0.5 mM glutamine to adjust the cell density to 1.5 × 10^5^/ml. The cells were plated at a density of 6 × 10^5^ cells/dish on 6-well poly-l-lysine precoated dishes with 5% carbon dioxide at 37 °C in a humidified atmosphere. The medium was changed every 3–4 days. Confluent cells were used for subsequent experiments at 80–90% confluence.

To mimic SAE model, cultured cells were stimulated with 1 μg/ml LPS with/without HRS for 24 h. Cells in the control group were treated with 0.9 normal saline. The cells were randomly divided into four groups: sham, sham + HRS, LPS, LPS + HRS.

In order to analyse the effects of different doses of HRS on cells,cultured cells were treated with equal volumes of HRS (0.2 mmol/L, 0.4 mmol/L, 0.6 mmol/L, GeneCare Water Treatment Co.; Ltd (Beijing, China) and normal saline solution for 48 h. Thereafter, a 100 μl of culture medium containing 10 μl cell count kit-8 (CCK-8, Meilunbio, Dalian China) was used to replace the medium and incubated for 1 h, thereafter cell viability was detected in each group. A microplate reader (Bio Tek) was used to measure the absorbancy at 450 nm.

### Animal model

All experiments were performed by the Guide for the Care and Use of Laboratory Animals (Ministry of Health, China); the protocol was approved by the Animal Care Committee of Southern Medical University. All rats were housed with free access to food and water under standard conditions, including humidity of 55–65%, a temperature of 21–27 °C and a 12 h light/dark cycle.

### Chemicals and preparations

Hydrogen-rich saline (HRS) was procured from GeneCare Water Treatment Co.; Ltd (Beijing, China). HRS was prepared according to the manufacturer’s instructions. Briefly, it was dissolved in normal saline for 6 h at 4 °C in a 0.4 Megapascal (MPA) to a supersaturated level using hydrogen producing apparatus. Hydrogen-rich saline was freshly prepared weekly to maintain a 0.6 mmol/L concentration. A needle-type hydrogen sensor (Unisense A/S) was used to monitor the hydrogen concentration. Gram-negative bacterium lipopolysaccharide (LPS) 055:B5 (Sigma-Aldrich. St. Louis, MO, USA) was first dissolved in 0.9% saline (0.3 ml) for the subsequent induction of the SAE model.

### SAE animal model

Forty-eight juvenile male (4–5 weeks old, weighing 100–120 g) Sprague–Dawley (SD) rats were obtained from Southern Medical University (SMU) Experimental Animal facility, SCXK (Yue) 2021-0041. The SD rats were randomly divided into sham (SH) group, sham + HRS group, lipopolysaccharide (LPS) group and LPS + HRS group. LPS was first dissolved in saline and then administered at 8 mg/kg to induce the SAE model. The rats in the HRS treatment group received a single dose of HRS dissolved in normal saline at 5 ml/kg injected intraperitoneally for 1 h following LPS according to their corresponding group assignment. An equal volume of normal saline was injected accordingly in the control group.

### Nissl staining

After HRS treatment following SAE induction, rats were anaesthetised with chloral hydrate and then sacrificed at 48 h post-SAE by transcardiac perfusion with saline followed by 4% paraformaldehyde in 0.1 M PBS solution. The brains were quickly removed and postfixed with 4% paraformaldehyde embedded in paraffin at 4 °C left overnight. Brain tissues were coronally sectioned with a thickness of 4 µm. The sliced specimen were hydrated in 0.1% cresyl violet for 2 min, then dehydrated in ethanol and cleared with xylene according to the manufacturer’s instructions (Beyotime Biotechnology (C0117). Afterwards, the stained slides were observed with a LEICA DM2500 microscope. Cell number was counted per high-power field and each high-power field was photographed in 6 different visual fields (0.6 mm^2^).

### Neurological Scores and survival analysis

Neurological function was assessed using a standard scoring system [39]: 0 = no apparent deficits, 1 = contralateral forelimb flexion, 2 = decreased grip of contralateral forelimb, 3 = contralateral circling if pulled by tail, 4 = spontaneous contralateral circling. Rats were followed for 48 h to measure the survival rate after induction of sepsis.

### Enzyme-linked immunosorbent assay (ELISA)

Serum and tissue supernatants were prepared according to the instructions. Venous blood drawn from rats were first centrifuged and the plasma collected and stored at − 80 °C for further analysis. The plasma levels of TNF-α, IL-1β and IL-10 were measured using a commercially available enzyme-linked immunosorbent assay (ELISA) kit (Cusabio Wuhan, China) according to the manufacturer’s instructions. Brain tissues were stored at − 80 °C and then were thawed by stepwise temperature increase (−20 °C and + 4 °C, respectively). Tissue supernatants were obtained from brain tissue by sonication in phosphate-buffered saline (PBS). Serum and tissue homogenate was then subjected to centrifugation at 3000 rpm for 10 min at + 4 °C. Serum and tissue homogenates of the brain tissue samples were used to determine the levels of TNF-α, IL-1β and IL-10. Microplate readers analysed absorbance values at different wavelengths (Biotek Instruments, Inc., Vermont, USA). The results are expressed as pg/ml.

### TUNEL staining

The brain tissue specimens were quickly removed and postfixed with 4% paraformaldehyde embedded in paraffin at 4 °C left overnight, and sectioned into 4 μm thickness slices. Apoptotic cells were visualized by in situ detection of DNA fragmentation (terminal deoxynucleotidyl transferase-mediated dUTP nick end labelling, TUNEL) according to the manufacturer’s instructions (Elabscience Biotechnology Co.; Ltd Wuhan, China). Five slices were selected randomly in each group and analyzed with a LEICA DM2500 microscope. The Apoptosis Index (AI) was calculated by Image J software. AI = the number of apoptotic cells / total number of cells × 100%. Slices of brain tissues were further analyzed by Hoechst 33258 (Beyotime Biotechnology) and visualized under fluorescence microscope to determine nuclei fragmentation of apoptotic cells.

### Mitochondrial function

#### MMP measurement

Mitochondrial membrane potential (MMP) was measured using JC1 dye (Beyotime Biotechnology) according to the manufacturer’s instructions. Briefly, the mitochondria were first isolated from brain tissues using a Tissue Mitochondrial isolation kit (Beyotime Biotechnology) according to the manufacturer’s instructions. Following the experiment, the isolated mitochondria were dyed with JC1 staining solution at 37 °C for 30 min, and the fluorescent properties changed from green to red when the level of MMP was high. Red fluorescent JC1 aggregates form in hyperpolarized membranes, whereas green fluorescent monomeric forms indicate membrane depolarization. The higher the ratio of red to green fluorescence, the more intact the mitochondrial membrane is.

#### ATP content detection

The ATP content was determined with the ATP Solarbio Life Sciences Assay kit according to the manufacturer’s instructions. Briefly, blood samples of rats were first centrifuged, and the plasma collected and stored ad − 80 °C. Brain tissue supernatants were extracted and centrifuged at 1000 g 4 °C for 10 min, and then transferred to an ice-cold EP tube. Subsequently, the APT content was measured in both plasma and homogenized lysates using a microplate reader (Biotek Instruments, Inc.).

#### Determination of mtROS

mtROS production was measured in brain tissue supernatants using the ROS-specific fluorescent probe (Applygen Technologies Inc.), and dichlorodihydrofluorescein diacetate (DCFHDA). The fluorescence intensity was measured in a fluorescent microplate reader (BioTek Instruments, Inc.). The fluorescence intensity was normalized to that of the control group.

#### Western blotting

Brain tissue specimens of rats were homogenized in ice-cold PBS, then frozen and stored at − 80 °C until analysis. Brain tissues were collected in lysis buffer (2% SDS, 1% Triton X-100, 50 mM Tris–HCl and 150 mM NaCl, pH 7.5), and a protease inhibitor cocktail (Roche, USA) for the detection of GFAP, IBA-1, Bcl-2 and Bax proteins by Western blotting. The samples were centrifuged at 14,000 × g for 15 min at 4 °C and the supernatants were collected for measurements of protein concentrations using the bicinchoninic acid (BCA) technique according to the manufacturer’s protocol. Protein (15 μg) lysates were separated by SDS-PAGE electrophoresis, transferred to nitrocellulose membrane and analyzed by conventional immunoblotting. Antibodies were diluted in a blocking solution containing TBS-T(150 mM NaCl, 8 mM K2HPO4, 1.99 mM KH2HPO4, 0.1% Tween) and the membranes incubated overnight at 4 °C with the following primary antibodies: GFAP (LOT. no. 59h2301), AIF1/IBA-1 (LOT. no. 52c1105), Bcl-2 (LOT. no. 11o9905) and Bax (LOT. no. 44q6915), all from Affinity Biosciences. Subsequently, the membranes were washed with TBST three times, followed by incubation with corresponding horseradish peroxidase-conjugated rabbit IgG secondary antibody (LOT. no. BST17A04A17B54, Boster) at 37 °C for 1 h. After washing with TBS-T three times, proteins were then probed with an ECL + Plus chemiluminescence reagent kit (Amersham) to visualize the signal followed by exposure to X-ray film. Gel-Pro Image Analyzer Software was used to analyze protein bands in SDS-PAGE. The density ratio represented the relative intensity of each band against GAPDH (LOT no. GR3316865-11, Abcam).

#### Immunohistochemistry and immunofluorescence staining

Brain tissues of rats were homogenized in ice-cold PBS and stored at -80 °C for further analysis. Isolated coronal Sects. (4 µm) were incubated with 3% hydrogen peroxide (H_2_O_2_) in PBS and incubated overnight with primary antibodies against GFAP, IBA-1 (1: 200; Affinity Biosciences). The sections were then treated with a secondary antibody (PV-9000, OriGENE, Beijing, China) according to the manufacturer’s instructions, and tissue sections were stained with diaminobenzidine (DAB) for 2 min to detect nuclear DNA. The sections were then visualized using a LEICA DM2500 microscope.

For immunofluorescence analysis, animals were perfused with PBS followed by 4% paraformaldehyde. After perfusion, the brain was removed and postfixed in a fixative solution for 4 h, placed in PBS containing 30% sucrose, and then stored at 4 °C. Brain sections were cut in 7 µm with a cryostat (LEICA RM2016) and processed for immunofluorescence. After blocking with 5% BSA at room temperature for 30 min, sections were then incubated overnight at 4 °C with primary antibodies against GFAP (LOT no. Gb12096) and IBA-1 (LOT no. gb12105). Sections were washed with 0.1 M PBS 3 times, 5 min each and incubated with HRS anti-goat secondary antibody (CY3, GB21303). After wash, immunofluorescence mounting buffer containing 4’, 6-diamidino-2-phenylindole (DAPI) (Servicebio, China) was used for covering and observed with a fluorescence microscope (NIKON Eclipse C1, Japan).

#### Electron microscopy

The brain tissue samples were harvested, cut into small pieces and fixed with 2% paraformaldehyde and 0.25% glutaraldehyde in phosphate buffer at 4 °C overnight. Sections were then washed with the same buffer and postfixed with 0.25% glutaraldehyde and 1% phosphate-buffered osmium tetroxide at 4 °C overnight. Samples were sliced into sections of about 60–80 μm prepared in EMBed812 resin (SP 90529-77-4) and mounted onto copper grids. After the fixation, a graded series of concentrations of ethanol (30, 50, 70, 90, 95 and 4 × 100% each for 20 min) was used to dehydrate and acetone for 15 min, and then samples were mounted onto copper wire mesh. The sections were stained with 2% uranyl acetate for 30 min at 4 °C, followed by staining in 2.6% lead citrate solution for 30 min at room temperature. The sections were observed using a transmission electron microscope (Hitachi, Ltd., Tokyo, Japan).

#### Statistical analysis

Data were presented as means ± SD. The survival rate was estimated using the Kaplan–Meier method, and the survival curves were determined by Log-rank (Mantel-Cox) test. Multiple comparisons between two groups were performed by ANOVA followed by the Turkey test. GraphPad Prism 6.0 software was used for all statistical tests. Values with p < 0.05 were considered significant.

## Results

### Effect of HRS on neurobehavioural function 48 h after sepsis induction

**Fig. 1:**
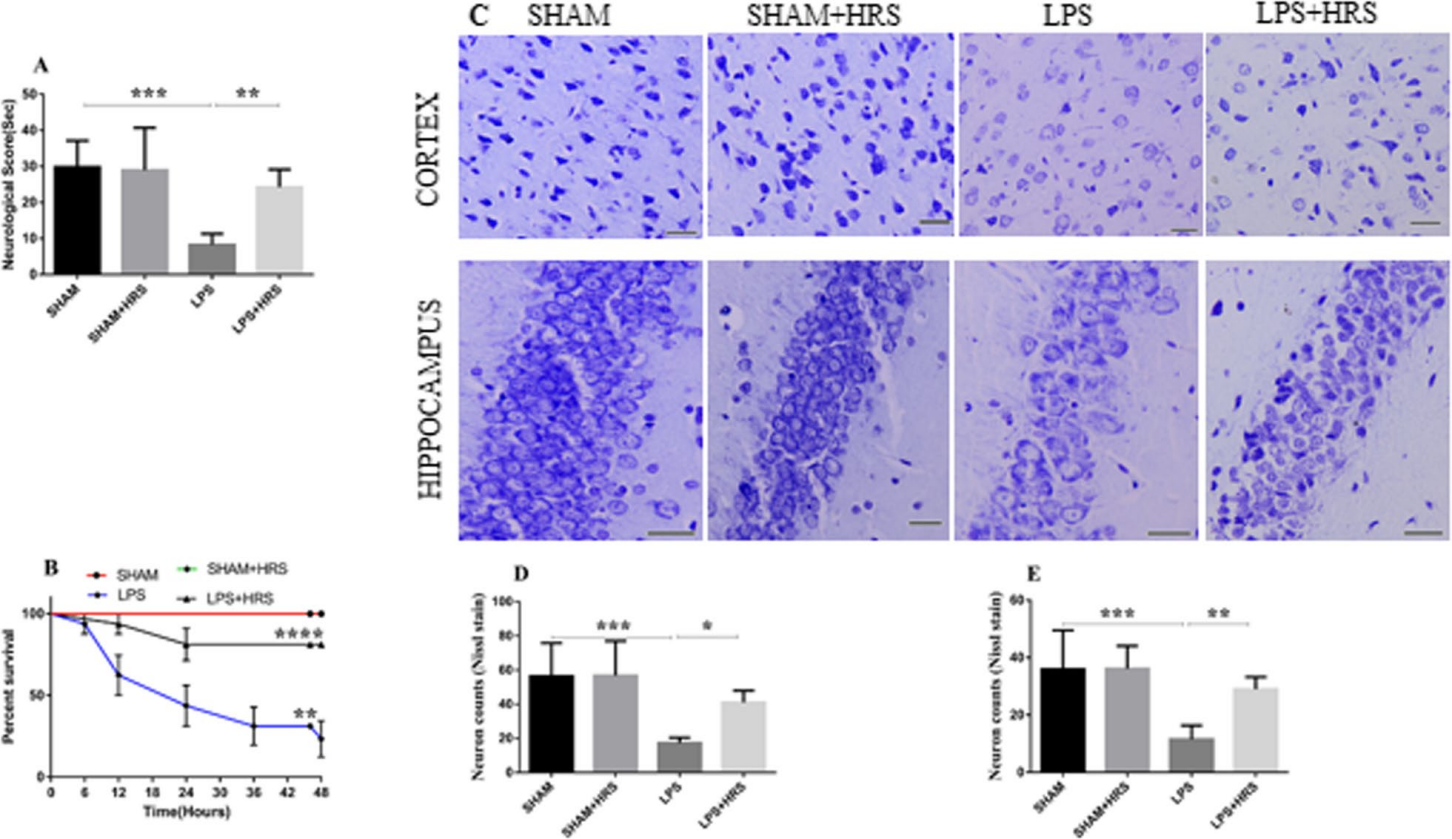
HRS treatment attenuated neurological score and neuronal injury in SAE rat model 48 h after LPS induction. **A**, **B** Statistical representation of neurological test and survival curves of septic rats in each group, respectively. **C** The photograph indicates decreased number of normal neurons stained with Nissl stain, showing disordered cellular structure with shrunken cytoplasm and perineuronal vacoulation with dark nuclei in the LPS group, which were not obvious in the HRS treated group in both the cortex and hippocampus, respectively (n = 5). Scale bar = 50 μm **D**, **E** Quantification of Nissl stained neuronal cells in the cortex **D** and hippocampus **E** of each group. All data are presented as mean ± SD ****p < 0.0001, ***p < 0.001, **p < 0.005, *p < 0.05. *HRS* Hydrogen-rich saline, *LPS* Lipopolysaccaride

The neurological function was assessed in HRS-treated rats with sepsis induced by LPS (Fig. 1A). As shown above, the neurological function was decreased in the LPS group compared to the sham-operated group, with a mean percentage of total points scored of 29.85 ± 7.32 and 8.27 ± 3.09%, respectively. This was significantly improved in the HRS-treated group, with a mean of 24.25 ± 4.88% (p < 0.05). The survival rate assessed at the end of the study period by the log-rank (Mantel-Cox) test showed a downward sloping of the survival curve in the LPS and HRS groups compared to the sham or sham + HRS group (Fig. 1B). However, the downward sloping of the survival curve was more obvious in the LPS group. The survival rate in the LPS-treated rats was 25.00%, while the survival rate in the HRS-treated group was 81.25% (p < 0.05). Additionally, all animals in the sham and sham + HRS groups survived during the experiment. This data indicates that HRS treatment could improve the survival rate in septic rats.

### HRS improves neuronal injury 48 h following LPS-induced sepsis

Nissl staining in brain tissues of septic rats showed dense and purple blue nuclei and abundant Nissl bodies in the cytoplasm of neurons in the sham-operated or sham + HRS group; whereas, in the LPS group there were increased neuronal loss, disordered and sparse neurons, blurred and/or absent Nissl bodies, tissue breakdown and neuronal vacuolization in both the cerebral cortex and hippocampus, while HRS treatment decreased neuron loss, tissue breakdown and vacuolization, and increased the number of normal neurons and Nissl bodies compared to the LPS-treated group thereby ameliorating neuronal injury in both cerebral cortex and hippocampus, as shown in Fig. 1C. Positive neuron number was also decreased in the LPS group of the cerebral cortex and hippocampus compared to the sham group (Fig. 1D, E). Conversely, this was increased after HRS administration in the cerebral cortex and hippocampus, the mean corrected normal neuronal cell count per high-power field in the HRS group was 41.00 ± 7.04 and 29.00 ± 4.30% compared to 17.33 ± 3.14 and 11.50 ± 4.85% in the LPS group, respectively (p < 0.05).

### HRS attenuates inflammatory mediators 48 h following LPS-induced sepsis in cultured cells and rats

**Fig. 2:**
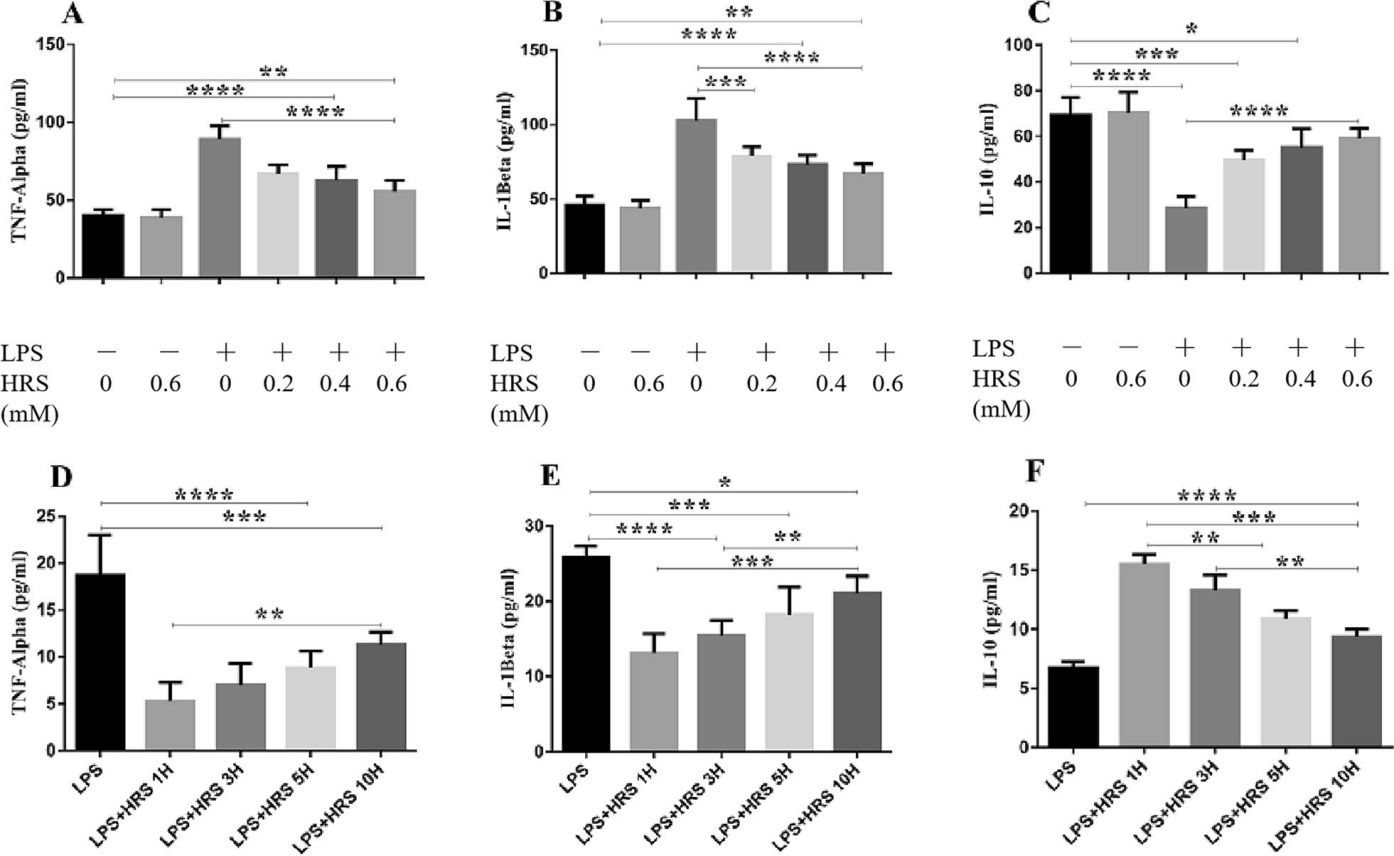
Effect of HRS on inflammatory cytokines expression in SH-SY5Y cells induced by LPS in each group. Cells were exposed to LPS and HRS medium and the supernatants were collected for detection of TNF-α, IL-1β and IL-10 in a concentration-dependent manner **A**–**C** and time interval-dependent manner **D**–**F** Data are presented as mean ± SD (n = 6) ****p < 0.0001, ***p < 0.001, **p < 0.005, *p < 0.05. *HRS* Hydrogen-rich saline, *LPS* Lipopolysaccharide, *TNF-α* Tumour necrosis factor alpha, *IL-1β* Interleukin-1 beta, *IL-10* Interleukin 10

We investigated the effects of HRS on inflammatory cytokines expression given at a various time interval following sepsis induction by ELISA assay. As shown in Fig. 2A–C, cells stimulated with LPS showed increased expression of TNF-α and IL-1β while IL-10 was decreased. HRS treatment decreased TNF-α and IL-1β and increased IL-10 expression levels at different time interval when compared to the LPS group (p < 0.05). However, HRS given as early as 1 h after LPS-induced sepsis was more effective in decreasing TNF-α and IL-1β expression levels and increasing IL-10 levels compared to other time intervals (p < 0.05). We further analyzed the effect of HRS on inflammatory cytokines expressions in cells stimulated with LPS in a concentration-dependent manner. The levels of TNF-α and IL-1β were elevated and IL-10 decreased in the LPS-treated cells compared to sham group (p < 0.05). Consequently, this effects was reversed in the HRS-treated cells where TNF-α and IL-1β expressions were decreased and increased IL-10 expression level in a time-dependent manner (Fig. 2D–F). These data suggest that HRS exerts its effectiveness in a concentration-dependent manner and that this effectiveness was more pronounced when given as early as 1 h after model induction.

Furthermore, the levels of inflammatory mediators were also estimated in rat serum and brain tissues of LPS-induced sepsis by ELISA assays. The levels of TNF-α and IL-1β were significantly elevated in the LPS group compared with the sham-operated or sham + HRS group in serum of septic rats, 18.65 ± 7.20 and 30.44 ± 9.15 vs. 6.03 ± 1.34 and 15.23 ± 2.12 pg/ml (p < 0.05), respectively, while HRS treatment ameliorated the excessive release of TNF-α and IL-1β in serum of rats, 8.64 ± 0.86 and 18.35 ± 1.45, respectively (Table 1). However, the expression of IL-10 was significantly decreased in the LPS group compared to the sham-operated group, 9.25 ± 2.89 vs. 16.78 ± 1.05 pg/ml, respectively; while the increased release of IL-10 induced by LPS was elevated in the HRS-treated group, 14.31 ± 2.00 pg/ml. Brain tissues of septic rats showed a similar effect, where TNF-α and IL-1β were increased (93.06 ± 15.59 and 104.50 ± 28.08 vs. 22.19 ± 2.07 and 28.93 ± 2.36 pg/ml, respectively), and decreased-10 expression levels (38.38 ± 7.29 vs. 65.19 ± 7.62 pg/ml) in the LPS group compared to the sham-operated group; this was reverted in the HRS-treated group (46.59 ± 7.65, 55.24 ± 6.54 and 57.74 ± 9.62 pg/ml, respectively) (p < 0.05, Table 2). This data indicates the effect of HRS in regulating neuroinflammation by decreasing pro-inflammatory mediators (TNF-α, IL-1β) and increasing anti-inflammatory mediators (IL-10) in septic rats.

**Table 1:** Systemic inflammatory cytokines expressions in serum of septic rats (mean ± SD)

	TNF-α^	IL-1β^	IL-10^
SHAM	6.03 ± 1.34	15.23 ± 2.12	16.78 ± 1.05
SHAM + HRS	6.02 ± 2.72	15.46 ± 2.50	16.57 ± 2.09
LPS	18.65 ± 7.20*	30.44 ± 9.15*	9.25 ± 2.89*
LPS + HRS	8.64 ± 0.86#	18.35 ± 1.45#	14.31 ± 2.01#

*HRS* Hydrogen-rich saline, *LPS* Lipopolysaccharide, *TNF-α* Tumour necrosis factor alpha, *IL-1β* Interleukin-1 beta, *IL-10* Interleukin 10

^ Measured in pg/ml; Data are presented as mean ± SD (n = 6)

*p < .0.05 vs. Sham group

#p < 0.05 vs. LPS group

**Table 2:** Inflammatory cytokines expression levels in brain tissues of septic rats (mean ± SD)

	TNF-α^	IL-1β^	IL-10^
SHAM	22.19 ± 2.07	28.93 ± 2.36	65.19 ± 7.62
SHAM + HRS	22.37 ± 9.29	29.42 ± 7.29	66.01 ± 10.10
LPS	93.06 ± 15.59*	104.50 ± 28.08*	38.38 ± 7.91*
LPS + HRS	46.59 ± 7.65*#	55.24 ± 6.54*#	57.74 ± 9.62#

*HRS* Hydrogen-rich saline, *LPS* Lipopolysaccharide, *TNF-α* Tumour necrosis factor alpha, *IL-1β* Interleukin-1 beta, *IL-10* Interleukin 10

^ measured in pg/ml; Data are presented as mean ± SD (n = 6)

*p < .0.05 vs. Sham group

#p < 0.05 vs. LPS group

### HRS improves neuronal apoptosis neurons 48 h after LPS-induced sepsis

**Fig. 3:**
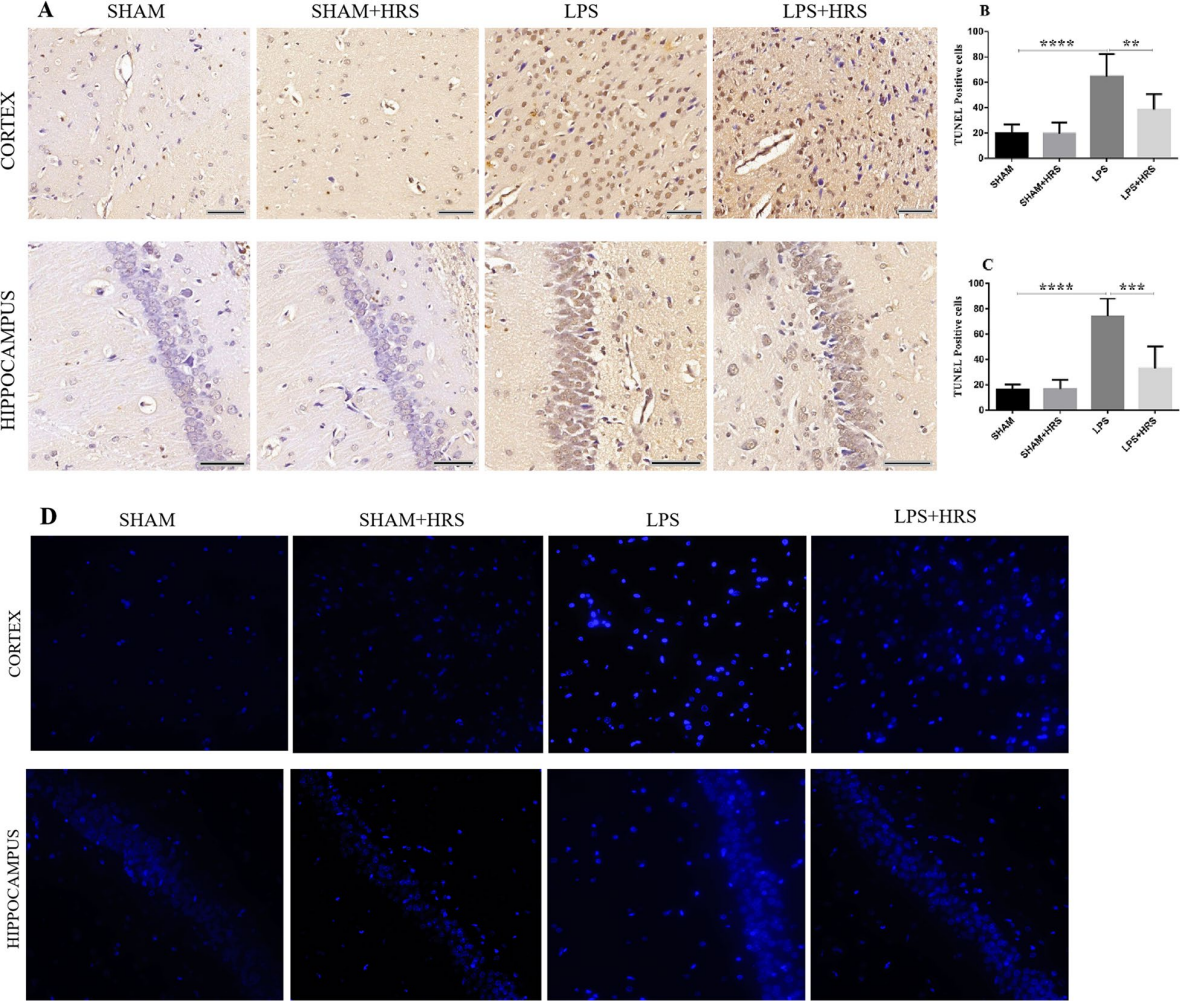
HRS effects in neuronal apoptosis in the cerebral cortex and hippocampus in all groups 48 h after LPS administration (n = 5). **A** TUNEL immunoreactive stained sections showed increased neuronal apoptotic cell death in the LPS group compared to sham group, which was significantly decreased in the HRS-treated group. Scale bar = 50 µm. **B**, **C** Representation of mean number of TUNEL positive cells in the cerebral cortex and hippocampus. **D** Apoptotic cells were further stained with Hoechst 33258 showing dense blue appearance of apoptotic nuclei or fragmented cells. Increased bright blue stain apoptotic cells were observed in the LPS group compared to sham group, which were significantly decreased in the HRS-treated group. Data are presented as mean ± SD ****p < 0.0001, ***p < 0.001, **p < 0.005

Recent studies have shown that systemic application of LPS is sufficient to cause neuronal cell death and diminished neuronal density in both the hippocampus and cortex [40]. We therefore assessed neuronal apoptotic cell death in septic rats by TUNEL staining. As shown in Fig. 3, the number of apoptotic cells was increased in the LPS-treated group compared to the sham-operated or sham + HRS group in both the cerebral cortex and hippocampus, characterized by brown stain apoptotic neuronal cells and shrunken morphology in the injured rat brain (Fig. 3A), these changes were improved in the HRS-treated group with less brown stained apoptotic cells (p < 0.05). The percentage of TUNEL-positive cells in LPS and HRS-treated groups was 64.67 ± 17.76 vs. 38.33 ± 12.47% and 74.17 ± 13.89 vs. 33.00 ± 17.34% in the cortex and hippocampus, respectively (Fig. 3B, C), (p < 0.05). This data suggest that HRS treatment can improve neuronal damage by decreasing the number of apoptotic cell death. Further analysis by Hoechst 33258 showed increased dense bright blue appearance of apoptotic nuclei or fragmented cells in the LPS group compared to the sham group. This bright blue appearance was decreased in the HRS-treated group both in the cerebral cortex and hippocampus (Fig. 3D).

### Effect of HRS on astrocytes and microglia activation in rats with LPS-induced sepsis

**Fig. 4:**
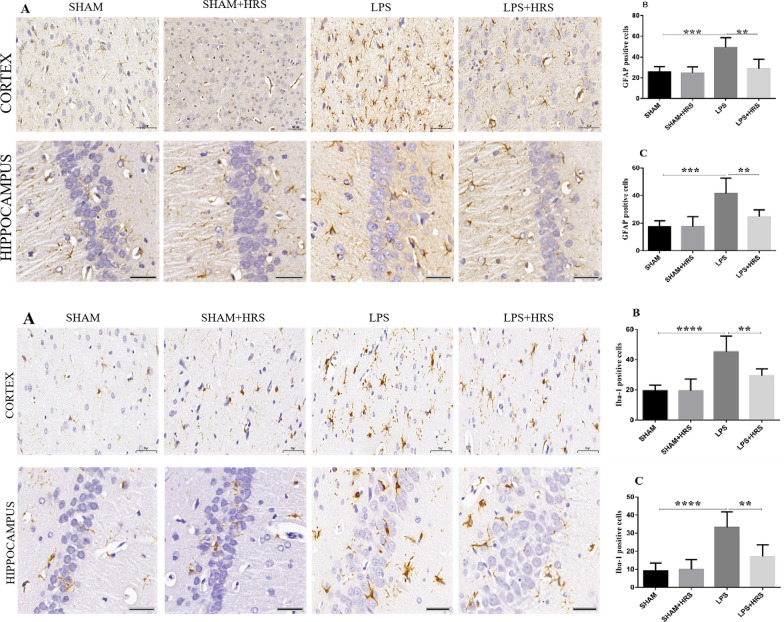
HRS treatment attenuated astrocyte and microglial activation in cerebral cortex and hippocampus of septic rats 48 h after LPS induction. The photographs present immunohistochemistry staining showing the number of GFAP(top diagram) and IBA-1 (bottom) Positive cells in each group, respectively (n = 6). Scale bar = 50 μm. **A**, **B** Quantification of GFAP and IBA-1positive cells in cerebral cortex and hippocampus in each group, respectively. All data are presented as mean ± SD ****p < 0.0001, ***p < 0.001, **p < 0.005. *HRS* Hydrogen-rich saline, *LPS* lipopolysaccharide, *GFAP* Glial fibrillary acidic protein, *IBA-1* Ionised calcium binding adaptor molecule 1

**Fig. 5:**
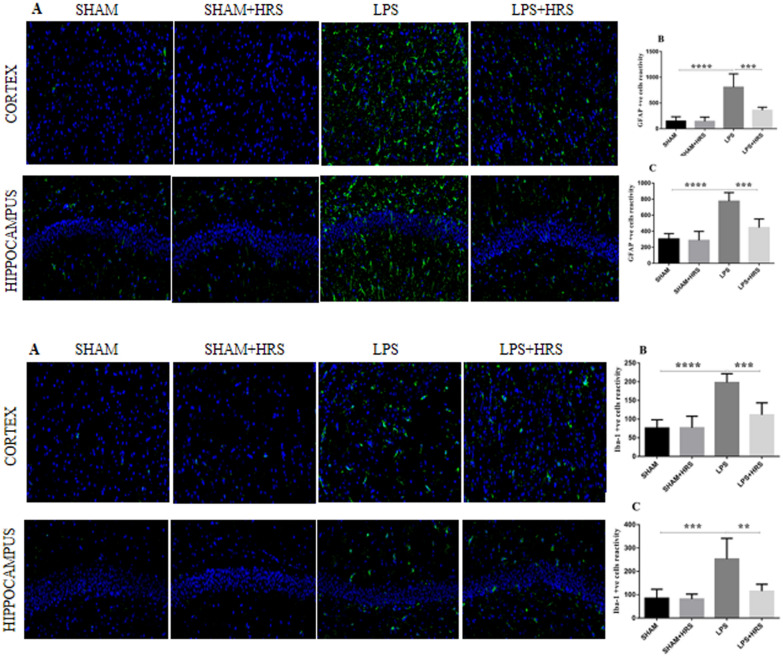
HRS treatment attenuated astrocyte and microglial activation in cerebral cortex and hippocampus of septic rats 48 h after LPS induction. Photographs represent astrocyte and microglial activation showing immunoreactivity of GFAP (top diagram) and IBA-1 (bottom) in cerebral cortex and hippocampus, respectively. Scale bar = 50 μm. **A**, **B** statistical representation of GFAP and IBA-1 showing the mean number of immunoreactive cells in cerebral cortex and hippocampus in each group, respectively (n = 6). All data are presented as mean ± SD ****p < 0.0001, ***p < 0.001, **p < 0.005. *HRS* Hydrogen-rich saline, *LPS* lipopolysaccharide, *GFAP* Glial fibrillary acidic protein, *IBA-1* Ionised calcium binding adaptor molecule 1

Immunohistochemistry analysis of brain tissues 48 h after LPS administration showed an increased number of GFAP (top diagram) and IBA-1 (bottom diagram) positive cells in the LPS-treated group compared to the sham-operated or sham + HRS group in the cerebral cortex and hippocampus (p < 0.05). Conversely, this was decreased in the HRS-treated group compared with the LPS-treated group (Fig. 4A–C; top: GFAP; bottom: IBA-1). The immunofluorescence analysis showed a significant increase in the number of GFAP and IBA-1 positive cells immunoreactivity in the LPS-treated group compared to the sham-operated group (p < 0.05). Similarly, this was decreased in the HRS-treated group compared with the LPS-treated group in both the cerebral cortex and hippocampus (Fig. 5A–C; top: GFAP; bottom: IBA-1). This result suggests that activated astrocytes and microglia in septic rats can be abrogated after HRS treatment.

### HRS alleviates mitochondrial dysfunction 48 h following LPS-induced sepsis in rats

**Fig. 6:**
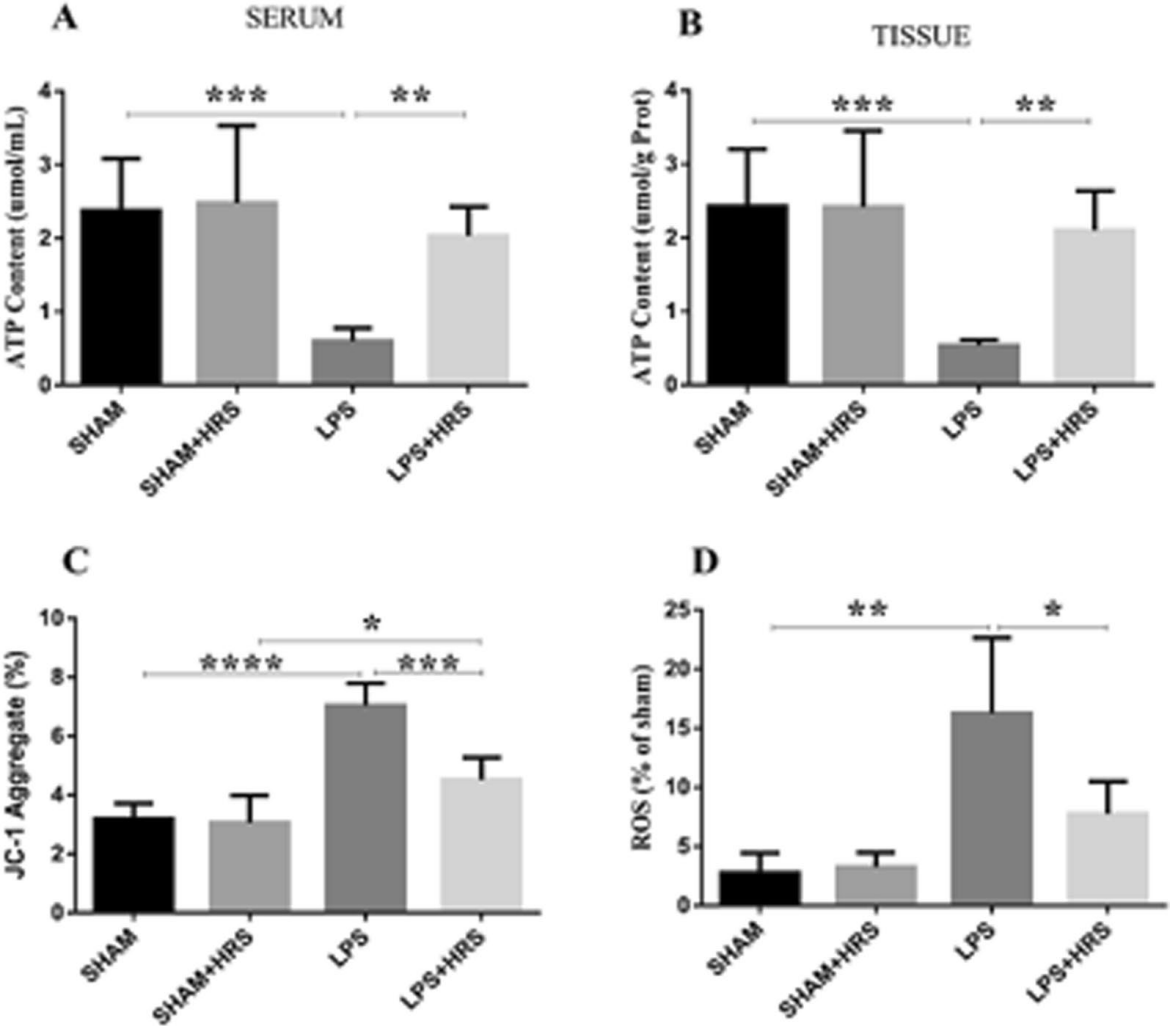
HRS treatment attenuated mitochondrial dysfunction in septic rats 48 h after LPS administration. **A** Measurement of MMP damage detected by JC-1 dye showing up-regulation of monomer/J-aggregate in the LPS group (implying increased MMP damage), while HRS treatment showed the reversed effects. **B**, **C** ATP content detection in serum and brain tissues of septic rats, depicting decreased ATP content in the LPS group, respectively; this was reversed in the HRS-treated group. **D** Mitochondrial ROS damage measured by DCFH-DA showing increased DCF fluorescence label in LPS group compared to sham group, which was decreased in the HRS-treated group (n = 6) All data are presented as mean ± SD ****p < 0.0001, ***p < 0.001, **p < 0.005, *p < 0.05. *HRS* Hydrogen-rich saline, *LPS* Lipopolysaccharide, *ATP* Adenosine triphosphate, *MMP* mitochondrial membrane potential, *DCFH-DA* Dichloro-dihydro-fluorescein diacetate, *ROS* Reactive oxygen species

Mitochondrial function was assessed through MMP, ATP content, and mtROS detection in brain tissues of septic rats. As shown in Fig. 6, the MMP damage was measured by JC-1 dye that showed reduced MMP (the monomer/J-aggregates value was upregulated) in the LPS-treated group compared to the sham group, 7.04 ± 0.75 vs. 3.20 ± 0.57%, respectively (p < 0.05). Whereas, the HRS-treated group exhibited a significant increase in MMP (the monomer/J-aggregates value was downregulated) compared to the LPS group, 4.52 ± 0.76 vs. 7.04 ± 0.75% (Fig. 6A). No statistical significance between sham and sham + HRS group. The ATP content was measured in serum and brain tissues of septic rats, which showed decreased ATP content in the LPS group compared to the sham group, 0.60 ± 0.18 vs. 2.38 ± 0.71 and 0.54 ± 0.08 vs. 2.43 ± 0.78 μmol/mL, respectively (p < 0.05), this was increased in the HRS-treated group in both serum and brain tissues 2.03 ± 0.40 and 2.10 ± 0.54 μmol/mL, respectively (Fig. 6B, C), (p < 0.05). No statistical significance between sham and sham + HRS group. Mitochondrial ROS damage was measured by DCFH-DA showing the increased release of mtROS in the LPS group compared to the sham-operated and sham + HRS groups, 16.25 ± 6.40 vs. 2.75 ± 1.71% (Fig. 6D), while the HRS-treated group exhibited a significant decrease in mtROS release in septic rats compared to the LPS-treated group, 7.75 ± 2.75 vs. 16.25 ± 6.40% (p < 0.05). These data suggest that HRS treatment ameliorated mitochondrial dysfunction and injury in septic rats induced by LPS.

### Effect of HRS on mitochondrial damage and ultrastructural disruption in LPS-induced septic rats

**Fig. 7:**
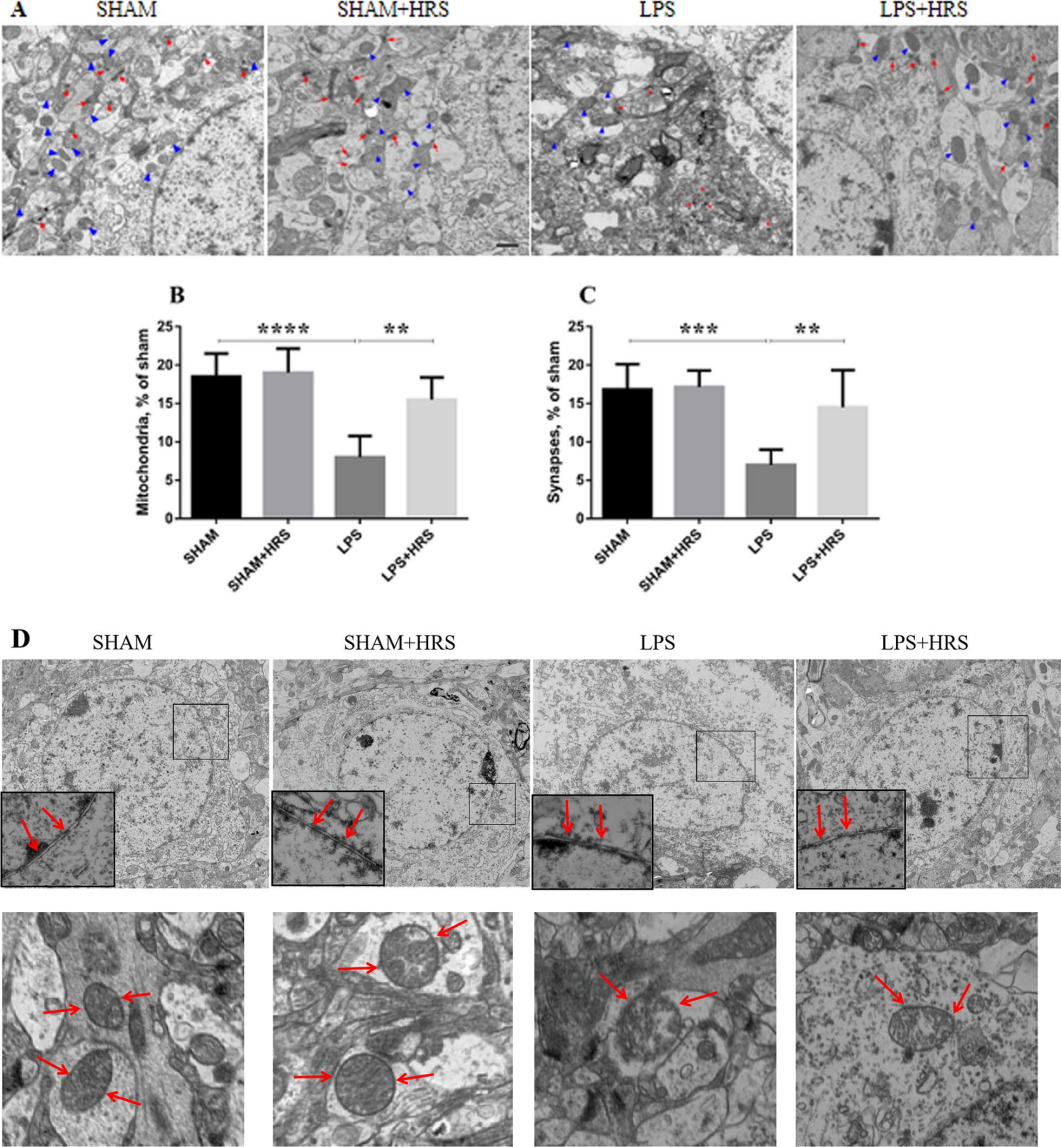
HRS treatment attenuated ultrastructural damage of mitochondria in brain tissues of septic rats 48 h after LPS challenge. **A** TEM micrographs indicate number of mitochondria (blue arrowheads) and synapses (red arrows) in each group, respectively. Scale bar = 50 μm. **B**, **C** statistical representation of the mean number of mitochondria and synapses in all groups (n = 5). **D** Graphical representation of mitochondrial ultrastructure depicting double nuclei membrane disruptions. The arrows showed intact and continuous mitochondrial membrane in the sham and HRS-treated groups, which was disrupted in the LPS group (inset & lower photographs). Upper panels = X4000, lower panels = X15000 magnifications. All data are presented as mean ± SD ****p < 0.0001, ***p < 0.001, **p < 0.005. *HRS* hydrogen-rich saline, *LPS* Lipopolysaccharide, *TEM* Transmission electron microscope

We assessed mitochondrial damage 48 h after LPS administration by transmission electron microscopy. The number of mitochondria and synapses was decreased in the LPS group compared to the sham or sham + HRS group (Fig. 7A–C), implying increased mitochondrial damage in septic rats induced by LPS. However, the number of mitochondria and synapses was increased in the HRS-treated group (p < 0.05) No statistical significance between sham and sham + HRS group. Further ultrastructural analysis by TEM showed disrupted and discontinuous mitochondrial nuclei membrane (red arrows, inset & lower photographs) in the LPS group compared to the sham or sham + HRS group showing an intact double nuclei membrane without disruption (Fig. 7D). The disrupted and discontinuous nuclei membrane of the mitochondria was improved in the HRS-treated group; implying the positive effect HRS has in maintaining intact mitochondrial structure.

### Effect of HRS on GFAP, IBA-1, Bcl-2 and Bax protein expression in rats with LPS-induced sepsis

**Fig. 8:**
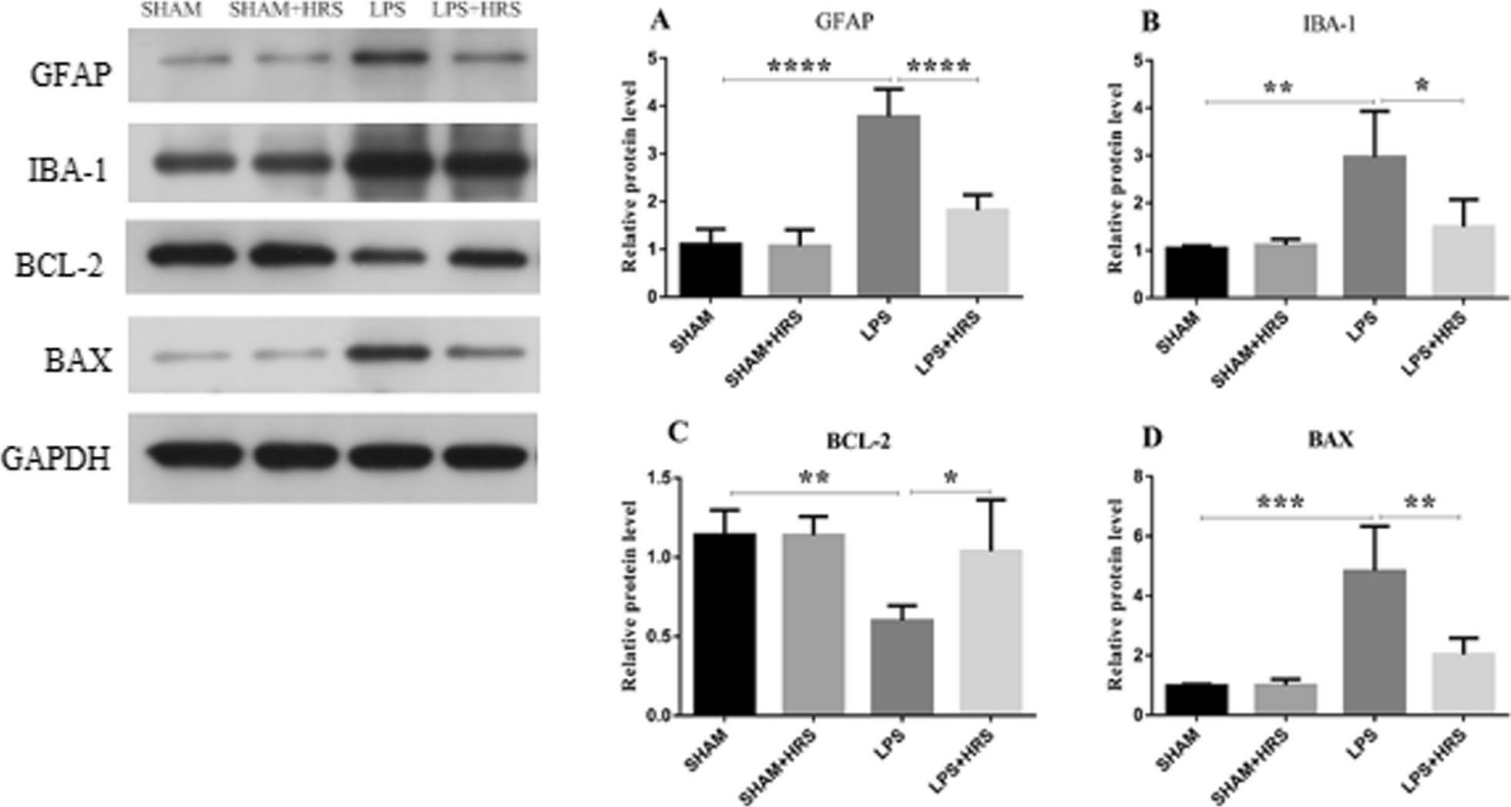
Effects of HRS treatment on protein expressions in septic rats 48 h after LPS challenge. **A** Representative images of protein expression levels by western blot analysis in each group. **B**–**E** Statistical representation of relative protein expressions of GFAP, IBA-1, BCL-2 and BAX; respectively. All data are presented as mean ± SD ****p < 0.0001, ***p < 0.001, **p < 0.005, *p < 0.05. *HRS* Hydrogen-rich saline, *LPS* Lipopolysaccharide, *GFAP* Glial fibrillary acidic protein, *IBA-1* Ionised calcium binding adaptor molecule 1, *BCL-2* B-cell lymphoma 2

Protein expressions of GFAP, IBA-1, Bcl-2 and Bax were analyzed in LPS-induced septic rats treated with HRS by western blot. As shown in Fig. 8A, B, the relative protein expression levels of GFAP and IBA-1 were markedly increased in the LPS-treated group compared to the sham-operated group (3.78 ± 0.58 vs 1.11 ± 0.32 and 2.97 ± 0.97 vs 1.05 ± 0.05%, respectively), while HRS treatment significantly reduced the protein expressions of GFAP and IBA-1 (1.82 ± 0.32 and 1.50 ± 0.57%, respectively) (p < 0.05). Bax was elevated in LPS-induced septic rats compared to the sham group (4.83 ± 1.51 vs 0.99 ± 0.05%). However, the Bcl-2 expression was reduced in the LPS-treated compared to the sham-operated group (0.60 ± 0.09 vs 1.14 ± 0.15%) (Fig. 8C, D). Conversely, Bax demonstrated a statistically significant reduction in the HRS-treated group compared to the LPS-treated group (2.04 ± 0.34 vs 4.83 ± 1.51%). Moreover, HRS treatment significantly increased Bcl-2 expression compared with the LPS group (1.04 ± 0.33 vs 0.60 ± 0.09%) (p < 0.05).

### Effects of HRS on cell viability and inflammatory cytokines expression in LPS-induced sepsis

**Fig. 9:**
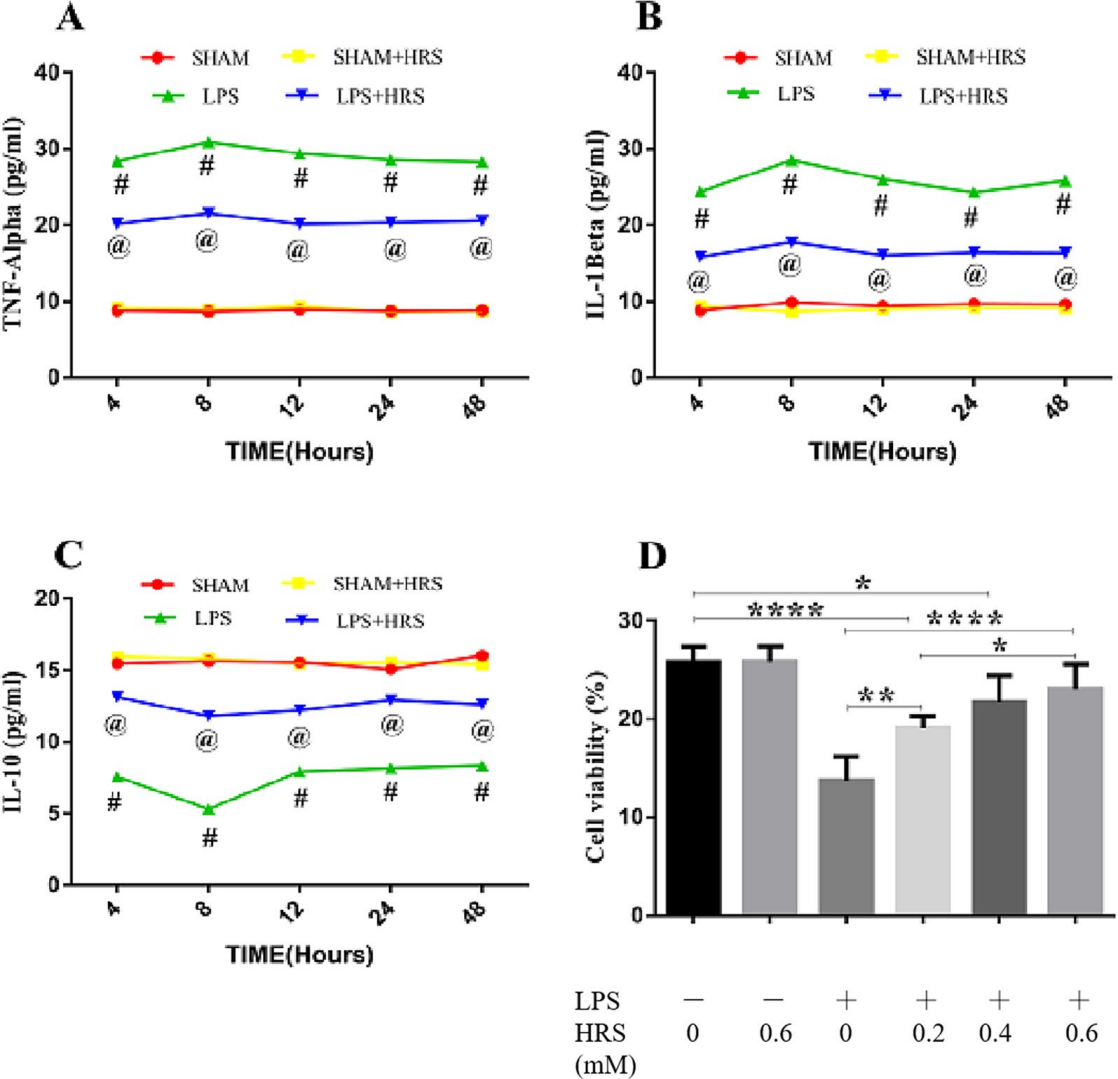
Effect of HRS on cell viability and inflammatory cytokines in SH-Y5Y cells treated with LPS in each group. Cells were stimulated with LPS for 4 h and replaced with HRS medium. At 48 h after sepsis induction cells were harvested and supernatants collected for TNF-α, IL-1β and IL-10 detection in a time-dependent manner **A**–**C** and cell viability by CCK-8 assay **D**. All data are presented as mean ± SD (n = 6) ****p < 0.0001, ***p < 0.001, **p < 0.005, *p < 0.05. *HRS* Hydrogen-rich saline, *LPS* Lipopolysaccharide, *TNF-α* Tumour necrosis factor alpha, *IL-1β* Interleukin-1 beta, *IL-10* Interleukin 10

In vitro analysis of inflammatory mediators were estimated in cultured cells stimulated with LPS by ELISA assays. The levels of TNF-α and IL-1β were elevated and IL-10 decreased in the LPS-treated cells compared to sham group (p < 0.05). Consequently, this effects was reversed in the HRS-treated cells where TNF-α and IL-1β expressions were decreased and increased IL-10 expression level in a time-dependent manner (Fig. 9A–C). We further investigated the effects of HRS on cell viability at different concentrations of HRS assessed by CCK-8 assays. As shown in Fig. 9D, we observed a decrease in cell viability in response to LPS stimulation. However, treatment with HRS showed increased cell viability in a concentration-dependent manner. These data suggest that HRS exerts its effectiveness in a concentration-dependent manner in septic rats.

## Discussion

In this study, we reported the neuroprotective effects of hydrogen-rich saline (HRS) in attenuating neuroinflammation, neuronal injury, improved survival rate, and improving mitochondrial dysfunction in the juvenile SAE rat model. This is also the first study to demonstrate the effects of HRS in the paediatric SAE model. SAE represents a challenge in the ICU with high mortality and morbidity but with few available treatment options other than systemic support and antibiotics that are sometimes associated with brain dysfunction in critically ill patients as a side effect [41], especially in children due to the complexity of their brain development and disease course.

The pathophysiology of SAE is multifactorial involving diffuse neuroinflammation, disrupted BBB, mitochondrial dysfunction, oxidative stress, excitotoxicity, and cerebral autoregulation impairment [11, 42, 43]. These pathogenetic mechanisms have similar characterisation both in adults and children, though their pathogenesis and clinical presentation might differ due to the evolving brain. For instance, systemic adaptive and immune responses following infections, the resistance of the immature brain to injury [44, 45], and functional BBB response after brain injury insults [46]. Most of these factors are said to be regulated by HRS, in part via neuroinflammatory attenuation, oxidative stress modulation, maintaining BBB integrity, ameliorating mitochondrial dysfunction, regulating astrocyte and microglia activation and suppressing NLRP3 inflammasome activation in adults SAE models [19, 25, 47].

Neuroinflammation results from the upregulation of pro-inflammatory cytokines (PICs) that are involved in microcirculatory dysfunction by potentially altering blood flow [11]. Upregulated PICs mediate SAE occurrence due to their direct correlation with BBB disruption, brain edema, neutrophil infiltration, astrocytosis, and apoptosis of brain cells [48]. The mRNA expression of TNF-α and its receptor, TNFR1 is also upregulated following LPS induction in the septic encephalopathy model [49]. Similarly, stimulated microglia and astrocytes by cytokines further produce other cytokines, chemokines, nitric oxide, excitatory amino acids, COX2, and reactive oxygen species (ROS), which are detrimental to the immature brain due to the enhanced vulnerability of maturing cells [50]. Our results showed that PICs, e.g., TNF-α and IL-1β were upregulated while anti-inflammatory cytokines, e.g., IL-10 was downregulated in septic rats induced by LPS thereby exacerbating neuroinflammation. However, HRS treatment attenuated neuroinflammation by reducing TNF-α and IL-1β expression levels and subsequently increasing IL-10 levels both in serum and brain tissues of septic rats. Previous studies have reported similar effects [47, 51]. A recent control trial also demonstrated the protective effect of HRS in healthy individuals by decreasing inflammatory response and increasing antioxidant capacity [52]. In vivo studies showed that reactive astrocytes (A1) phenotype is also induced by TNF-α and IL-1β after being stimulated by LPS [53], the current study also reported similar results in cultured cells, thus it is conceivable that there exist a synergy between astrocytes activation and upregulation of pro-inflammatory mediators (TNF-α and IL-1β) during sepsis. In essence, decreasing A1 reactive astrocytes is crucial in curtailing neuroinflammation and astrocytes activation. It is therefore plausible to state that the mechanistic effects of HRS is through balancing and maintaining activation of inflammatory mediators as demonstrated above where TNF-α and IL-1β were downregulated and IL-10 upregulated, suppressing A1 reactive astrocytes and microglia activation in septic rat model. Additionally, we demonstrated that HRS given at early onset of sepsis induction is more effective in reducing TNF-α and IL-1β than when given at later time point, though more studies are needed to validate this effect.

Neuronal sensitivity from increased levels of nitric oxide (NO) produced by activated microglia can exacerbate neuronal apoptosis [9]. Certain chemokines also promote neuronal apoptosis, as evidenced by increased upregulation of Ccl2 or Cxcl2 protein levels in the PLS model, resulting in hippocampal neuronal apoptosis, thus supporting a direct role of these chemokines in neuronal death [54]. Another study reported that postnatal day 1 (p1) is more vulnerable to neurotoxicity than postnatal day 2 (p2) in septic rat model induced either induced by hypoxia–ischemia, LPS or both, due to the immaturity of neuronal cells [44]. In our present study, both neuronal injury (evidenced by increased number of damaged neurons, dissolved cellular structure and dark nuclei, shrunken cytoplasm and perineuronal vacuolisation) and neuronal apoptotic cell death were increased after LPS administration in SAE rats, while HRS treatment abrogated neuronal injury and apoptotic neuronal cell death both in the cerebral cortex and hippocampus. HRS enhanced the efficiency of the defense response evidenced by improved resolution of neuroinflammation in sickness behavior induced by LPS [29], as well as attenuation of neuronal apoptosis in early brain injury of SAH rat model [55]. We also reported improved neurological function at 48 h following LPS induction, as well as an improved survival rate after HRS administration. Other studies have also demonstrated the effect of H2 in attenuating neurobehavioral deficits and neuronal apoptosis in a neonatal hypoxia–ischemia rat model [38, 56]. Based on these results and ours, it is conceivable that HRS protective mechanism in septic models is probably via counteracting pro-apoptotic mediators’ activation and ameliorating neuronal injury.

Mitochondria as the powerhouse of cellular metabolism, is responsible for over 90% of the total body oxygen consumption and ATP generation via oxidative phosphorylation [57]. Mitochondria play a vital role in neuronal functions, and alteration of mitochondrial dynamics including fission and fusion can have deleterious effects. In an SAE model, LPS stimulation led to a loss of mitochondrial membrane potential, propagation of dynamin-related protein 1 (Drp1) and p53 recruitment with subsequent initiation of cell death pathways [58]. Decreased mitochondrial ATP generation is caused by increased release of ROS and NO, which in turn induce neuronal apoptosis by releasing cytochrome C [59]. In addition, activation of reactive nitrogen species (RNS), NO and ROS can inhibit complexes I and IV of the electron transport chain (ETC), which disrupt mitochondrial function that is implicated in SAE pathogenesis [9, 60]. Excessive production of ROS during sepsis exacerbates mitochondrial damage and decrease in ATP concentration, and subsequent depletion of cellular/energy metabolism, resulting in bioenergetic failure. Our present study demonstrated that the mitochondrial membrane potential (MMP) and ATP content were decreased in septic rats while ROS production was increased. Increased release of ROS after LPS induction resulted in mitochondrial dysfunction and directly or indirectly inhibiting mitochondrial respiratory chain complexes vital for normal cellular metabolism. Damaged mitochondria further impaired it ability to generate ATP, thus altering the bioenergetic status of cellular function. However, this excess release of ROS was abrogated after HRS treatment, thus improving mitochondrial dysfunction. ATP content is vital for maintaining constant supply of energy needed by the body. ATP concentration was reported to decrease in non-survivors of septic patient attributed to increase rise of AMP resulting in imbalance in ATP turnover, thus decreased ATP production [57]. Our present study demonstrated similar results where ATP content was decreased in septic rats that were conversely enhanced after HRS administration. Similarly, HRS improved MMP, further highlighting it effect in improving mitochondrial dysfunction. Interestingly, reactive astrocytes (A1) result in less formation of synapses and that even those that have formed are weaker compared to those produced by healthy resting astrocytes [53]. A recent study has demonstrated that single or multiple systemic injection of LPS result in global loss of synapses [40]. Our results further showed that the number of mitochondria and synapses following the LPS challenge were decreased. Thus, inhibiting A1 reactive astrocytes can have positive effect in increasing synaptic formation as well as maintaining strong and healthy synapses, which our results has demonstrated after HRS treatment. Similarly, mitochondrial biogenesis is also disrupted due to exacerbated PICs activation; and our results showed that the mitochondrial membrane was disrupted after LPS administration, resulting in ultrastructural damage to mitochondrial biogenesis. Other studies have reported similar results [4]. Furthermore, mitochondrial dysfunction due to uncontrolled leakage of electrons from the electron transfer chain can be mitigated after HRS administration, thus improving mitochondrial energy metabolism [61].

Recent studies showed that astrocytes and microglial cells are involved across SAE pathomechanistic spectrum, with inflammatory activation occurring mainly in microglial cells [62]. Microglia activation involved two phenotypes, M1 cells that produce PICs and ROS and M2 cells that produce anti-inflammatory mediators that play neuroprotective effect and tissue repair ability [62, 63]. Activated microglia deteriorates BBB integrity subsequently enhancing ROS release, which leads to brain dysfunction [48]. Studies have shown that microglia depletion during severe sepsis development is associated with early exacerbation of the brain and systemic inflammation in the sepsis model [64]. Similarly, astrocytes, which control homeostasis and catabolism, also have two forms; reactive astrogliosis triggers nervous tissue damage by attracting immune cells specifically to the injured region and facilitates their extravasation and tissue infiltration [65]. In addition, astrocytes have been reported to restore neuronal mitochodrial membrane potential that result in increased mitochondrial content [66]. in vivo studies have shown that reactive microglia are needed to induce A1 reactive astrocytes in LPS-treated cells [53]. Thus, astrocytes activation is exacerbated by reactive microglia, both are implicated in the pathogenesis of sepsis. Our present study demonstrated that LPS induced increased activation of astrocyte and microglia in septic rats as shown by elevated expressions of GFAP and Iba-1 in both cerebral cortex and hippocampal neurons, causing neurotoxicity. HRS administration decreased activation of these markers, thus ameliorating astrogliosis and microglial activation. Further analysis by immunofluorescence staining showed increased immunoreactivity of GFAP and Iba-1 in the cerebral cortex and hippocampus that was reverted after HRS treatment, highlighting the possible mechanistic action of HRS in modulating astrocytes and microglial activation implicated in the SAE pathogenesis. Another study reported similar findings where HRS treatment decreases M1 polarization thereby regulating microglia activation in a septic rat model [47]. At protein level, both GFAP and Iba-1 were downregulated in the HRS group when determined by western blot, suggesting it molecular actions in regulating these proteins. Though, further studies are needed to explore its targeted molecular function, our results have shown that HRS is crucial in maintaining mitochondrial function, modulating inflammatory mediators and regulating astrocytes and microglia activation.

A recent study showed that early fluid resuscitation and inhalation of 2% hydrogen could decrease oxidative damage and inhibit the overexpression of inflammatory factors, as well as downregulating the expression of proapoptotic protein Fas, and up-regulate the expression of anti-apoptotic protein Bcl2 in septic shock rats [67]. Our results also showed the effect of HRS in regulating apoptotic mediators, where Bcl-2 was downregulated and upregulated Bax expression levels in septic rats, while HRS treatment exerted the opposite effect in regulating these mediators at molecular level.

We demonstrated in this study that early HRS administration after the onset of sepsis is more favourable in exerting its effectiveness in regulating inflammatory response and improving mitochondrial dysfunction than when given at a later time point. This decrease in HRS effectiveness is attributed to delayed administration of HRS compounded with increased SAE severity. However, this trend of effectiveness warrants more studies to explore this phenomenon. Secondly, the present study did not assess the long-term effect of HRS in improving neurological function and memory impairment, as reported in adult SAE models [68, 69]. Although our current study does not provide the exact mode of action of HRS, a possible sequence of events from exposure to HRS to protection of sepsis-associated encephalopathy could be postulated as follow: HRS could exert it’s action on mitochondria first through its anti-oxidative stress function, followed by anti-apoptotic activity through inhibition of the intrinsic apoptosis pathways such as inhibition of the release of cytochrome C and the related cascade, resulting in increase of pro-survival molecules such as AkT and phosphorylated AkT (p-AkT) and activation of mTOR pathway leading to inhibition of microglia activation and attenuation of inflammation. Naturally, the mechanism(s) underlying HRS neuroprotective effects in attenuating neuroinflammation and modulating astrocyte and microglia activation need further elucidation in paediatric SAE models. Nevertheless, our present study has shown the potential mechanistic effects of HRS in ameliorating inflammatory response; inhibit astrocyte and microglia activation, neuronal injury, neuronal apoptosis, and mitochondrial dysfunction, thus postulating its application in paediatric SAE as a promising therapeutic agent.

## Data Availability

Not applicable.

## Abbreviations

ATP: Adenosine triphosphate
BBB: Blood–brain barrier
BCL-2: B-cell lymphoma 2
CNS: Central nervous system
COX2: Cyclooxygenase 2
DCFH-DA: Dichloro-dihydro-fluorescein diacetate
ETC: Electron transfer chain
GAPDH: Glyceraldehyde phosphate dehydrogenase
GFAP: Glial fibrillary acidic protein
H2: Molecular hydrogen
HRS: Hydrogen-rich saline
IBA-1: Ionised calcium binding adaptor molecule 1
ICU: Intensive care unit
IL-1β: Interleukin-1 beta
IL-10: Interleukin 10
LPS: Lipopolysaccharide
MMP: Mitochondrial membrane potential
MODS: Multiple organ dysfunction syndromes
mtROS: Mitochondrial reactive oxygen species
NLRP3: Nucleotide-binding domain-like receptor protein 3
NO: Nitric oxide
PICs: Pro-inflammatory cytokines
RNS: Reactive nitrogen species
ROS: Reactive oxygen species
SAE: Sepsis-associated encephalopathy
TEM: Transmission electron microscope
TNF-α: Tumour necrosis factor alpha

## References

[1] Matics TJ, Sanchez-Pinto LN (2017). Adaptation and validation of a pediatric sequential organ failure assessment score and evaluation of the sepsis-3 definitions in critically Ill children. JAMA Pediatr.

[2] Schlapbach LJ, Straney L, Bellomo R, MacLaren G, Pilcher D (2018). Prognostic accuracy of age-adapted SOFA, SIRS, PELOD-2, and qSOFA for in-hospital mortality among children with suspected infection admitted to the intensive care unit. Intensive Care Med.

[3] Weiss SL, Peters MJ, Alhazzani W, Agus MSD, Flori HR, Inwald DP, Nadel S, Schlapbach LJ, Tasker RC, Argent AC, Brierley J, Carcillo J, Carrol ED, Carroll CL, Cheifetz IM, Choong K, Cies JJ, Cruz AT, De Luca D, Deep A, Faust SN, De Oliveira CF, Hall MW, Ishimine P, Javouhey E, Joosten KFM, Joshi P, Karam O, Kneyber MCJ, Lemson J, MacLaren G, Mehta NM, Møller MH, Newth CJL, Nguyen TC, Nishisaki A, Nunnally ME, Parker MM, Paul RM, Randolph AG, Ranjit S, Romer LH, Scott HF, Tume LN, Verger JT, Williams EA, Wolf J, Wong HR, Zimmerman JJ, Kissoon N, Tissieres P (2020). Surviving sepsis campaign international guidelines for the management of septic shock and sepsis-associated organ dysfunction in children. Intensive Care Med.

[4] Zhao YZ, Gao ZY, Ma LQ, Zhuang YY, Guan FL (2017). Research on biogenesis of mitochondria in astrocytes in sepsis-associated encephalopathy models. Eur Rev Med Pharmacol Sci.

[5] Sanz D, D'Arco F, Robles CA, Brierley J (2018). Incidence and pattern of brain lesions in paediatric septic shock patients. Br J Radiol.

[6] Yang Y, Liang S, Geng J, Wang Q, Wang P, Cao Y, Li R, Gao G, Li L (2020). Development of a nomogram to predict 30 day mortality of patients with sepsis-associated encephalopathy: a retrospective cohort study. J Intensive Care.

[7] Sandquist MK, Clee MS, Patel SK, Howard KA, Yunger T, Nagaraj UD, Jones BV, Fei L, Vadivelu S, Wong HR (2017). High frequency of neuroimaging abnormalities among pediatric patients with sepsis who undergo neuroimaging. Pediatr Crit Care Med.

[8] Kaur J, Singhi P, Singhi S, Malhi P, Saini AG (2016). Neurodevelopmental and behavioral outcomes in children with sepsis-associated encephalopathy admitted to pediatric intensive care unit: a prospective case control study. J Child Neurol.

[9] Nwafor DC, Brichacek AL, Mohammad AS, Griffith J, Lucke-Wold BP, Benkovic SA, Geldenhuys WJ, Lockman PR, Brown CM (2019). Targeting the blood-brain barrier to prevent sepsis-associated cognitive impairment. J Cent Nerv Syst Dis.

[10] Helbing DL, Böhm L, Witte OW (2018). Sepsis-associated encephalopathy. CMAJ.

[11] Ziaja M (2013). Septic encephalopathy. Curr Neurol Neurosci Rep.

[12] Huang L, Peng S, Li R, Xie D, Huang D (2020). Fulminant encephalopathy in a child with hyperferritinemic sepsis: a case report. BMC Neurol.

[13] Czempik PF, Pluta MP, Krzych ŁJ (2020). Sepsis-associated brain dysfunction: a review of current literature. Int J Environ Res Public Health.

[14] Ebersoldt M, Sharshar T, Annane D (2007). Sepsis-associated delirium. Intensive Care Med.

[15] Ehler J, Petzold A, Wittstock M, Kolbaske S, Gloger M, Henschel J, Heslegrave A, Zetterberg H, Lunn MP, Rommer PS, Grossmann A, Sharshar T, Richter G, Nöldge-Schomburg G, Sauer M (2019). The prognostic value of neurofilament levels in patients with sepsis-associated encephalopathy—a prospective, pilot observational study. PLoS ONE.

[16] Chaudhry N, Duggal AK (2014). Sepsis associated encephalopathy. Adv Med.

[17] Hadem J, Hafer C, Schneider AS, Wiesner O, Beutel G, Fuehner T, Welte T, Hoeper MM, Kielstein JT (2014). Therapeutic plasma exchange as rescue therapy in severe sepsis and septic shock: retrospective observational single-centre study of 23 patients. BMC Anesthesiol.

[18] Spapen H, Nguyen DN, Troubleyn J, Huyghens L, Schiettecatte J (2010). Drotrecogin alfa (activated) may attenuate severe sepsis-associated encephalopathy in clinical septic shock. Crit Care.

[19] Barancik M, Kura B, LeBaron TW, Bolli R, Buday J, Slezak J (2020). Molecular and cellular mechanisms associated with effects of molecular hydrogen in cardiovascular and central nervous systems. Antioxidants (Basel).

[20] Hu Q, Zhou Y, Wu S, Wu W, Deng Y, Shao A (2020). Molecular hydrogen: a potential radioprotective agent. Biomed Pharmacother.

[21] Iida A, Nosaka N, Yumoto T, Knaup E, Naito H, Nishiyama C, Yamakawa Y, Tsukahara K, Terado M, Sato K, Ugawa T, Nakao A (2016). The clinical application of hydrogen as a medical treatment. Acta Med Okayama.

[22] Ren JD, Wu XB, Jiang R, Hao DP, Liu Y (2016). Molecular hydrogen inhibits lipopolysaccharide-triggered NLRP3 inflammasome activation in macrophages by targeting the mitochondrial reactive oxygen species. Biochim Biophys Acta.

[23] Yang M, Dong Y, He Q, Zhu P, Zhuang Q, Shen J, Zhang X, Zhao M (2020). Hydrogen: a novel option in human disease treatment. Oxid Med Cell Longev.

[24] Xie K, Liu L, Yu Y, Wang G (2014). Hydrogen gas presents a promising therapeutic strategy for sepsis. Biomed Res Int.

[25] Qiu P, Liu Y, Zhang J (2019). Recent advances in studies of molecular hydrogen against sepsis. Int J Biol Sci.

[26] Muramatsu Y, Ito M, Oshima T, Kojima S, Ohno K (2016). Hydrogen-rich water ameliorates bronchopulmonary dysplasia (BPD) in newborn rats. Pediatr Pulmonol.

[27] Bai X, Liu S, Yuan L, Xie Y, Li T, Wang L, Wang X, Zhang T, Qin S, Song G, Ge L, Wang Z (2016). Hydrogen-rich saline mediates neuroprotection through the regulation of endoplasmic reticulum stress and autophagy under hypoxia-ischemia neonatal brain injury in mice. Brain Res.

[28] Shao A, Wu H, Hong Y, Tu S, Sun X, Wu Q, Zhao Q, Zhang J, Sheng J (2016). Hydrogen-rich saline attenuated subarachnoid hemorrhage-induced early brain injury in rats by suppressing inflammatory response: possible involvement of NF-κB pathway and NLRP3 inflammasome. Mol Neurobiol.

[29] Spulber S, Edoff K, Hong L, Morisawa S, Shirahata S, Ceccatelli S (2012). Molecular hydrogen reduces LPS-induced neuroinflammation and promotes recovery from sickness behaviour in mice. PLoS One.

[30] Ohsawa I, Ishikawa M, Takahashi K, Watanabe M, Nishimaki K, Yamagata K, Katsura K, Katayama Y, Asoh S, Ohta S (2007). Hydrogen acts as a therapeutic antioxidant by selectively reducing cytotoxic oxygen radicals. Nat Med.

[31] Li GM, Ji MH, Sun XJ, Zeng QT, Tian M, Fan YX, Li WY, Li N, Yang JJ (2013). Effects of hydrogen-rich saline treatment on polymicrobial sepsis. J Surg Res.

[32] Yao L, Chen H, Wu Q, Xie K (2019). Hydrogen-rich saline alleviates inflammation and apoptosis in myocardial I/R injury via PINK-mediated autophagy. Int J Mol Med..

[33] Li D, Ai Y (2017). Hydrogen saline suppresses neuronal cell apoptosis and inhibits the p38 mitogen-activated protein kinase-caspase-3 signaling pathway following cerebral ischemia-reperfusion injury. Mol Med Rep.

[34] Sobue S, Yamai K, Ito M, Ohno K, Ito M, Iwamoto T, Qiao S, Ohkuwa T, Ichihara M (2015). Simultaneous oral and inhalational intake of molecular hydrogen additively suppresses signaling pathways in rodents. Mol Cell Biochem.

[35] Wang L, Yin Z, Wang F, Han Z, Wang Y, Huang S, Hu T, Guo M, Lei P (2020). Hydrogen exerts neuroprotection by activation of the miR-21/PI3K/AKT/GSK-3β pathway in an in vitro model of traumatic brain injury. J Cell Mol Med.

[36] Chen H, Mao X, Meng X, Li Y, Feng J, Zhang L, Zhang Y, Wang Y, Yu Y, Xie K (2019). Hydrogen alleviates mitochondrial dysfunction and organ damage via autophagy-mediated NLRP3 inflammasome inactivation in sepsis. Int J Mol Med.

[37] Iketani M, Ohsawa I (2017). molecular hydrogen as a neuroprotective agent. Curr Neuropharmacol.

[38] Ke H, Liu D, Li T, Chu X, Xin D, Han M, Wang S, Wang Z (2020). Hydrogen-rich saline regulates microglial phagocytosis and restores behavioral deficits following hypoxia-ischemia injury in neonatal mice via the akt pathway. Drug Des Devel Ther.

[39] Liu Y, Liu W, Sun X, Li R, Sun Q, Cai J, Kang Z, Lv S, Zhang JH, Zhang W (2011). Hydrogen saline offers neuroprotection by reducing oxidative stress in a focal cerebral ischemia-reperfusion rat model. Med Gas Res.

[40] Manabe T, Rácz I, Schwartz S, Oberle L, Santarelli F, Emmrich JV, Neher JJ, Heneka MT (2021). Systemic inflammation induced the delayed reduction of excitatory synapses in the CA3 during ageing. J Neurochem.

[41] Mazeraud A, Righy C, Bouchereau E, Benghanem S, Bozza FA, Sharshar T (2020). Septic-associated encephalopathy: a comprehensive review. Neurotherapeutics.

[42] Chen J, Shi X, Diao M, Jin G, Zhu Y, Hu W, Xi S (2020). A retrospective study of sepsis-associated encephalopathy: epidemiology, clinical features and adverse outcomes. BMC Emerg Med.

[43] Catarina AV, Branchini G, Bettoni L, De Oliveira JR, Nunes FB (2021). Sepsis-associated encephalopathy: from pathophysiology to progress in experimental studies. Mol Neurobiol.

[44] Brochu ME, Girard S, Lavoie K, Sébire G (2011). Developmental regulation of the neuroinflammatory responses to LPS and/or hypoxia-ischemia between preterm and term neonates: an experimental study. J Neuroinflammation.

[45] James Anitha, Patel Vaishali (2014). Hypoxic ischaemic encephalopathy. Paediatrics And Child Health.

[46] Zhang W, Zhu L, An C (2020). The blood brain barrier in cerebral ischemic injury—disruption and repair. Brain Hemorrhages.

[47] Zhuang X, Yu Y, Jiang Y, Zhao S, Wang Y, Su L, Xie K, Yu Y, Lu Y, Lv G (2020). Molecular hydrogen attenuates sepsis-induced neuroinflammation through regulation of microglia polarization through an mTOR-autophagy-dependent pathway. Int Immunopharmacol.

[48] Ren C, Yao RQ, Zhang H, Feng YW, Yao YM (2020). Sepsis-associated encephalopathy: a vicious cycle of immunosuppression. J Neuroinflammation.

[49] Alexander JJ, Jacob A, Cunningham P, Hensley L, Quigg RJ (2008). TNF is a key mediator of septic encephalopathy acting through its receptor, TNF receptor-1. Neurochem Int.

[50] Bartha AI, Foster-Barber A, Miller SP, Vigneron DB, Glidden DV, Barkovich AJ, Ferriero DM (2004). Neonatal encephalopathy: association of cytokines with MR spectroscopy and outcome. Pediatr Res.

[51] Chen H, Xie K, Han H, Li Y, Liu L, Yang T, Yu Y (2015). Molecular hydrogen protects mice against polymicrobial sepsis by ameliorating endothelial dysfunction via an Nrf2/HO-1 signaling pathway. Int Immunopharmacol.

[52] Sim M, Kim CS, Shon WJ, Lee YK, Choi EY, Shin DM (2020). Hydrogen-rich water reduces inflammatory responses and prevents apoptosis of peripheral blood cells in healthy adults: a randomized, double-blind, controlled trial. Sci Rep.

[53] Liddelow SA, Guttenplan KA, Clarke LE, Bennett FC, Bohlen CJ, Schirmer L, Bennett ML, Münch AE, Chung WS, Peterson TC, Wilton DK, Frouin A, Napier BA, Panicker N, Kumar M, Buckwalter MS, Rowitch DH, Dawson VL, Dawson TM, Stevens B, Barres BA (2017). Neurotoxic reactive astrocytes are induced by activated microglia. Nature..

[54] Wolff S, Klatt S, Wolff JC, Wilhelm J, Fink L, Kaps M, Rosengarten B (2009). Endotoxin-induced gene expression differences in the brain and effects of iNOS inhibition and norepinephrine. Intensive Care Med.

[55] Hong Y, Shao A, Wang J, Chen S, Wu H, McBride DW, Wu Q, Sun X, Zhang J (2014). Neuroprotective effect of hydrogen-rich saline against neurologic damage and apoptosis in early brain injury following subarachnoid hemorrhage: possible role of the Akt/GSK3β signaling pathway. PLoS ONE.

[56] Wang P, Zhao M, Chen Z, Wu G, Fujino M, Zhang C, Zhou W, Zhao M, Hirano SI, Li XK, Zhao L (2020). Hydrogen gas attenuates hypoxic-ischemic brain injury via regulation of the MAPK/HO-1/PGC-1a pathway in neonatal rats. Oxid Med Cell Longev.

[57] Brealey D, Brand M, Hargreaves I, Heales S, Land J, Smolenski R, Davies NA, Cooper CE, Singer M (2002). Association between mitochondrial dysfunction and severity and outcome of septic shock. Lancet.

[58] Haileselassie B, Joshi AU, Minhas PS, Mukherjee R, Andreasson KI, Mochly-Rosen D (2020). Mitochondrial dysfunction mediated through dynamin-related protein 1 (Drp1) propagates impairment in blood brain barrier in septic encephalopathy. J Neuroinflammation.

[59] Cotena S, Piazza O (2012). Sepsis-associated encephalopathy. Transl Med UniSa.

[60] Heming N, Mazeraud A, Verdonk F, Bozza FA, Chrétien F, Sharshar T (2017). Neuroanatomy of sepsis-associated encephalopathy. Crit Care.

[61] Tian Y, Zhang Y, Wang Y, Chen Y, Fan W, Zhou J, Qiao J, Wei Y (2021). Hydrogen, a novel therapeutic molecule, regulates oxidative stress, inflammation, and apoptosis. Front Physiol.

[62] Moraes CA, Zaverucha-do-Valle C, Fleurance R, Sharshar T, Bozza FA, d'Avila JC (2021). Neuroinflammation in sepsis: molecular pathways of microglia activation. Pharmaceuticals (Basel).

[63] Michels M, Steckert AV, Quevedo J, Barichello T, Dal-Pizzol F (2015). Mechanisms of long-term cognitive dysfunction of sepsis: from blood-borne leukocytes to glial cells. Intensive Care Med Exp.

[64] Michels M, Ávila P, Pescador B, Vieira A, Abatti M, Cucker L, Borges H, Goulart AI, Junior CC, Barichello T, Quevedo J, Dal-Pizzol F (2019). Microglial cells depletion increases inflammation and modifies microglial phenotypes in an animal model of severe sepsis. Mol Neurobiol.

[65] Shulyatnikova T, Verkhratsky A (2020). Astroglia in sepsis associated encephalopathy. Neurochem Res.

[66] English K, Shepherd A, Uzor NE, Trinh R, Kavelaars A, Heijnen CJ (2020). Astrocytes rescue neuronal health after cisplatin treatment through mitochondrial transfer. Acta Neuropathol Commun.

[67] Liu W, Shan LP, Dong XS, Liu XW, Ma T, Liu Z (2013). Combined early fluid resuscitation and hydrogen inhalation attenuates lung and intestine injury. World J Gastroenterol.

[68] Zhuang K, Zuo YC, Sherchan P, Wang JK, Yan XX, Liu F (2020). Hydrogen inhalation attenuates oxidative stress related endothelial cells injury after subarachnoid hemorrhage in rats. Front Neurosci.

[69] Jiang Y, Zhang K, Yu Y, Wang Y, Lian N, Xie K, Yu Y (2020). Molecular hydrogen alleviates brain injury and cognitive impairment in a chronic sequelae model of murine polymicrobial sepsis. Exp Brain Res.

